# Screening of immunotherapy-related genes in bladder cancer based on GEO datasets

**DOI:** 10.3389/fonc.2023.1176637

**Published:** 2023-05-18

**Authors:** Xiaolong Liu, Xinxin Li, Qihui Kuang, Hongbo Luo

**Affiliations:** ^1^ School of Medicine, Wuhan University of Science and Technology, Wuhan, China; ^2^ Department of Urology, Wuhan Third Hospital and Tongren Hospital of Wuhan University, Wuhan, China; ^3^ Department of Urology, The Second Hospital of Huangshi, Huangshi, China; ^4^ Department of Urology, Renmin Hospital of Wuhan University, Wuhan, China

**Keywords:** bladder cancer, biomarker, immune cell infiltration, immunotherapy, abscopal effects

## Abstract

**Background:**

As one of the most prevalent genitourinary cancers, bladder cancer (BLCA) is associated with high morbidity and mortality. Currently, limited indicators are available for early detection and diagnosis of bladder cancer, and there is a lack of specific biomarkers for evaluating the prognosis of BLCA patients. This study aims to identify critical genes that affect bladder cancer immunity to improve the diagnosis and prognosis of bladder cancer and to identify new biomarkers and targets for immunotherapy.

**Methods:**

Two GEO datasets were used to screen differentially expressed genes (DEGs). The STRING database was used to construct a protein-protein interaction network of DEGs, and plug-in APP CytoHubba in Cytoscape was used to identify critical genes in the network. GO and KEGG analyses explored the functions and pathways of differential gene enrichment. We used GEPIA to validate the expression of differential genes, their impact on patient survival, and their relationship to clinicopathological parameters. Additionally, hub genes were verified using qRT-PCR and Western blotting. Immune infiltration analysis and multiple immunohistochemistry reveal the impact of Hub genes on the tumor microenvironment.

**Result:**

We screened out 259 differential genes, and identified 10 key hub genes by the degree algorithm. Four genes (ACTA2, FLNA, TAGLN, and TPM1) were associated with overall or disease-free survival in BLCA patients and were significantly associated with clinical parameters. We experimentally confirmed that the mRNA and protein levels of these four genes were significantly decreased in bladder cancer cells. Immunoassays revealed that these four genes affect immune cell infiltration in the tumor microenvironment; they increased the polarization of M2 macrophages.

**Conclusion:**

These four genes affect the tumor microenvironment of bladder cancer, provide a new direction for tumor immunotherapy, and have significant potential in the diagnosis and prognosis of bladder cancer.

## 1 Introduction

Bladder cancer (BLCA) is a common neoplastic disease in the urinary tract. Annually, more than 500,000 new bladder cancer cases diagnosed worldwide, resulting in more than 200,000 deaths (1). It is accountable for approximately 90-95 percent of all cases of urothelial carcinoma (2). The degree of bladder wall invasion defines the categorization of BLCA, which may be either invasive or non-invasive. More than 15% of patients with non-invasive BLCA progress to invasive BLCA after recurrence (3). Surgical resection, neoadjuvant chemotherapy, intravesical treatment, radiotherapy, and immune checkpoint inhibitors (ICIs) are conventional therapeutic approaches to bladder cancer (4). The most commonly used diagnostic methods for bladder cancer contain imaging tests, cystoscopy, and urine cytology (5). According to the study, cystoscopy is the only reliable method for diagnosis and post-treatment monitoring of bladder cancer (6). However, cystoscopy, as an invasive test, is not only a poor experience, but also costly and complicated to operate, and can increase the risk of urinary tract infection (7). In contrast, urine cytology is relatively simple and easy to accept. American Urological Association (AUA) recommend using urine cytology in specific circumstances. Unfortunately, despite the high specificity (approximately 86%) of cytopathology, the low sensitivity (48%) of urine cytology restricts its application (8), and it performs poorly in low-grade tumors (9). Recently, FDA has approved several urinary tests for diagnosis and monitoring of bladder cancer, including NMP22, ImmunoCyt/uCyt+, UroVysion (fluorescent *in situ* hybridization), and bladder tumor antigen (BTA) (10). However, even though recent research demonstrated their good outcomes in the context of NMIBC, there is limited evidence to prove their effect on the situation of initial diagnosis (11). In addition, several new urine tests are being tested, and in a large prospective study, ADX-BLADDER (MCM5) (12) showed higher sensitivity than urine cytology in the detection of high-grade bladder cancer, and EpiCheck (13), Xpert Bladder (14) and Cx-bladder (15) were highly valuable in ruling out recurrence of bladder cancer. Their practical use value in clinical practice needs to be further studied. Therefore, it makes sense to discover new possible markers for bladder cancer, which would help with early diagnosis, prognosis evaluation, and recurrence monitoring.

In this research, microarray analysis was used to detect and process the bladder cancer gene chip expression profile datasets downloaded from the GEO database. The datasets GSE3167 and GSE188715 contain 130 samples: 22 normal and 108 tumor samples. Using Perl and R, we obtained 259 DEGs, which were then analyzed with GO enrichment and KEGG pathway enrichment. Following the identifying potential genes, the GEPIA, TIMER, ACLBI, GeneMANIA and Metascape were consulted for survival analysis, co-expression analysis, immune related analysis and pathological staging analysis.

## 2 Materials and methods

### 2.1 Datasets and DEGs identification

The datasets obtained from studies in the GEO database which fulfilled the requirements listed below: (1) research involving the use of bladder cancer tissue samples; (2) research involving the use of containing information about the platform; (3) research involving the use of neighboring normal tissues as controls. Using the criteria as mentioned above, two datasets, GSE3167 (16) and GSE188715 (17), were identified and downloaded from the GEO database. The platform of the GSE3167 dataset is the Affymetrix Human Genome U133A Array. The platform of the GSE188715 is DNBSEQ-G400 (Homo sapiens). Using the R packages GEO Query and Limma R to analyze and identify GEO datasets, DEGs were successfully identified, with |fold change (FC)| > 2 and *p* < 0.05 being required. Venn diagram made by FunRich_3.1.4 (18). FunRich is an interactive tool that compares lists of Venn charts to identify genes that exhibit distinct levels of expression in each of the datasets above.

### 2.2 Analysis of GO, KEGG pathways and protein-protein interaction network

DAVID (Database for Annotation, Visualization and Integrated Discovery) 2021 database (https://david.ncifcrf.gov) (19) was used to identify and evaluate GO (Gene Ontology) terms (http://geneontology.org/) and KEGG (Kyoto Encyclopedia of Genes and Genomes) (https://www.genome.jp/). The ggplot2 (Version 3.3.6) (20) was adopted to visualize the analysis findings from DAVID. The threshold standard for substantial enrichment set at *p* < 0.05. STRING (Search Tool for the Retrieval of Interaction Gene/Proteins) (Version 11.5) (21) is an online tool used for PPI network analysis of DEGs, with *p* < 0.05 and the interaction scores of more than 0.7. STRING (Version 11.5) integrates known and anticipated interactions between more than 20 billion proteins across many animals, including Homo sapiens, allows the database to cover 67.6 million proteins in 14,094 different organisms. The Cytoscape (Version 3.9.1) (22) was then used to process that information to visualize the PPI network. The CytoHubba (23) is a plug-in, and used to determine the critical node genes of the PPI network.

### 2.3 Differential expression analysis and survival analysis

GEPIA (http://gepia.cancer-pku.cn/index.html) (24) is a dynamic website server with data from TCGA and GTEx, including 8587 normal samples and 9736 tumor samples. GEPIA offers a variety of user-configurable capabilities, including the examination of differential expression, gene detection, analysis based on clinical stages or cancer types, dimensionality reduction analysis, survival analysis, and correlation analysis. Using GEPIA, we examined putative differential genes in patients with bladder tumors for differential expression, disease-free survival, and overall survival.

### 2.4 TIMER, CIBERSORT, ACLBI, GeneMANIA Metascape, and TISCH2

TIMER (https://cistrome.shinyapps.io/timer/) (25) contains information about the abundance of immune cells infiltration in different types of tumors. Using TIMER, we analyzed the abundance of immune cells in the immune microenvironment of bladder cancer. The CIBERSORT algorithm assessed the abundance of immune cells in tumor samples. Based on the levels of gene expression, the cancer samples were separated into two groups; high and low expression groups are shown in red and blue, respectively. ACLBI (https://www.aclbi.com/static/index.html#/) (26) allows researchers to assess the differential expression, lymph node metastases, tumor stage, and other clinical characteristics using data from TCGA transcriptome and clinical patients. GeneMANIA (http://genemania.org/) (27) can predict the function of genes by indexing 2830 association networks with 660554667 connections mapped to 166691 genes from 9 species. Metascape (https://metascape.org) (28) can identify statistically enriched pathways and construct PPI networks. We used Metascape to explore co-expressed neighbor genes and identified the enrichment of pathways for neighbor genes. TISCH2 (http://tisch.comp-genomics.org/) is a tumor microenvironment-focused scRNA-seq database that offers extensive cell type annotation at the single-cell level, allowing for the investigation of the tumor microenvironment in various cancer types.

### 2.5 Cell culture

Normal bladder epithelial cells used in this study were SV-HUC-1 cultured in F-12K medium and bladder cancer cell line 5637 cultured in PMI-1640 medium. 1% penicillin-streptomycin solution and 10% fetal bovine serum were added to the culture medium. Both cell lines obtained from IMMOCELL (Xiamen, Fujian, China). All cells were grown in an atmosphere containing 5% CO2 at 37°C.

### 2.6 qRT-PCR

Trizol reagent was used to isolate RNA from SV-HUC-1 and 5637 cells (Thermo Fisher Scientific), and cDNA produced on the basis of the instructions provided by the kit manufacturer. qRT-PCR carried out using a StepOnePlusTM Real-Time PCR System (Applied Biosystems) with an SYBR Green-based reagent. (Qiagen, USA). The primer sequences were as follows: ACTA2, forward: GAGGGAAGGTCCTAACAGCC, reverse: GCTTCACAGGATTCCCGTCT; FLNA, forward: GGTCACGGGCTAGGTGC, reverse: CCGTCCTCATTCTCCACCAC; TAGLN, forward: AGGAATTGATGGAAACCACCG, reverse: ATGTCTGGGGAAAGCTCCTTG; TMP1, forward: TGAGCTCTCAGAAGGCCAAG, reverse: TCAGCTTGTCGGAAAGGACC.

### 2.7 Western blotting

Normal bladder cells and bladder cancer cells lysed using a Lysis Buffer that formulated with phosphatase inhibitors and a protease inhibitor cocktail. The BCA Protein Assay Kit was then used to determine the total quantity of protein. And polyacrylamide gel with a sodium dodecyl sulfate concentration of 10% was used to separate 20 to 30 g of each protein before putting it on a membrane made of polyvinylidene fluoride. The first blocking of membranes carried out in milk at a concentration of 5% for an hour, and then primary antibodies against ACTA2 [Catalog: A17910; ABclonal Technology], FLNA [Catalog: A16376; ABclonal Technology], TAGLN [Catalog: A6760; ABclonal Technology], TPM1 [Catalog: A1157; ABclonal Technology], CD163 [Catalog: A22619; ABclonal Technology] and β‐Actin [Catalog: AC006; ABclonal Technology]) were incubated with them. After being scanned by two-color infrared imaging equipment (Odyssey, LI-COR, USA), the blots examined with the ImageJ software. Finally, according to the instructions, cytoplasmic and nucleoprotein were separated using nuclear and cytoplasmic protein separation reagents. The Lysis Buffer was from Servicebio, the nuclear and cytoplasmic extraction reagent, phosphatase and protease inhibitor obtained from Thermo Fisher Scientific.

### 2.8 Multiple immunohistochemistry

mIHC staining performed on BLCA tissue samples. Before inhibiting endogenous peroxidase and incubating the antibody, in an autoclave, tissue sections were dewaxed and treated with Tris-EDTA pH 7.8 antigen retrieval solution for 5 minutes at 121°C. To perform multiplex fluorescence immunohistochemistry (mIHC), antibody staining had to be carried out in a series of sequential steps. These procedures comprised inhibiting peroxidase, applying the primary antibody, detecting with secondary horseradish peroxidase (HRP)-conjugated antibody, detecting with a fluorescent dye, and using a microwave to remove attached antibodies. In each experiment, three different antibodies stained using three different circulations. The slides mounted in an antifade solution after being counterstained with diamidino-2-phenylindole (DAPI). Bladder cancer tissue obtained from Shanghai Biochip Co, Ltd (Shanghai Biochip Co., Ltd, #HBlaU050CS01).

### 2.9 Statistical analysis

The qRT-PCR and Western blotting outcomes provided as mean ± SEM (Standard Error of Mean). The results between the two groups compared using t-test and conducted by GraphPad 6.0 (GraphPad Software, Inc.). It was deemed statistically significant if *p* < 0.05. In our study, all experiments performed three times.

## 3 Results

### 3.1 DEGs identification and Construction of PPI networks

**Figure 1:**
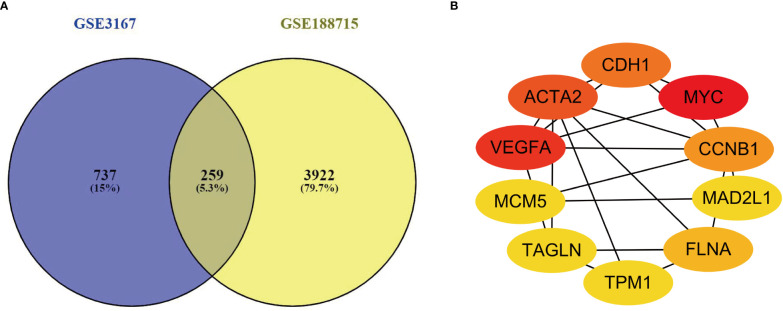
**(A)** There are a total of 259 DEGs identified from the GSE3167 and GSE188715. **(B)** Ten genes were found with the most connections. The redder the color, the higher the connection.

This investigation used two datasets (GSE3167 and GSE188715). GSE3167 comprised 9 normal samples and 51 tumor samples, while GSE188715 featured 13 normal samples and 57 tumor samples. VENNY2.1 found 259 common differential genes in the two included datasets (**Figure 1A**).

PPI networks were built for the 259 genes with differential expression, and Cytoscape 3.9.1 software and STRING database used to screen which genes were hub genes in the networks. The STRING-built PPI network had a total of 259 nodes, and it considered to be high correlated with the interaction score > 0.7. We identified the 10 genes with the most connections between them by applying Cytoscape version 3.9.1 (**Figure 1B**).

### 3.2 Enrichment analysis of DEGs

**Figure 2:**
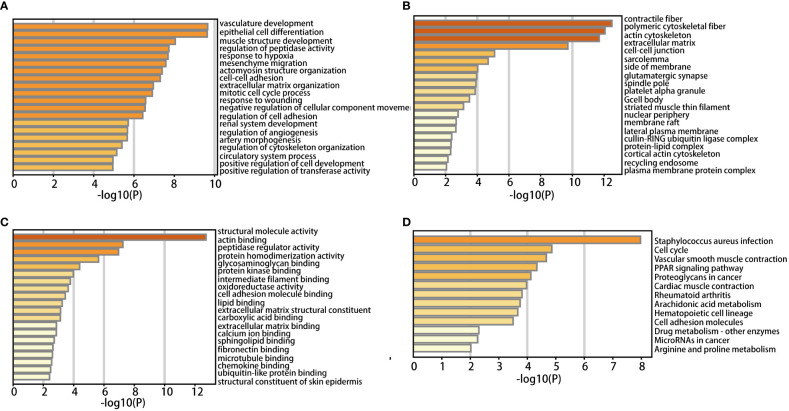
GO enrichment analysis in BP **(A)**, CC **(B)**, MF **(C)**, KEGG pathway enrichment **(D)** of DEGs.

DAVID was used to analyze functional enrichment. GO enrichment analysis mainly includes biological process (BP), cell composition (CC), and molecular function (MF). We found that DEGs were enriched primarily on BP, including vascular development, the structure of the epithelial cell differentiation, muscle development, the adjustment of the peptide enzyme activity, lack of oxygen reaction, interstitial migration, actomyosin structure organization, intercellular adhesion and extracellular matrix organization, mitotic cell cycle process, and response to injury (**Figure 2A**). CC analysis included contractile fiber, polymeric cytoskeletal fiber, actin cytoskeleton, extracellular matrix, intercellular junctions, myofibrillary membranes, one side of membranes, glutamate synapses, and spindle poles (**Figure 2B**). MF analysis included structural molecular activity, actin binding, peptidase regulatory activity, protein homologous dimerization activity, glycosaminoglycan binding, protein kinase binding, intermediate filament binding, oxidoreductase activity, cell adhesion molecule binding and lipid binding (**Figure 2C**).

According to KEGG pathway analysis, 259 DEGs primarily enriched in 13 pathways: staphylococcus aureus infection, cell cycle, vascular smooth muscle contraction, PPAR, proteoglycan in cancer, myocardial contraction, rheumatoid arthritis, arachidonic acid metabolism, hematopoietic cell lineage, cell adhesion molecules, drug metabolism - other enzymes, microRNAs in cancer, arginine and proline metabolism (**Figure 2D**).

### 3.3 Analysis of the expression and survival

**Figure 3:**
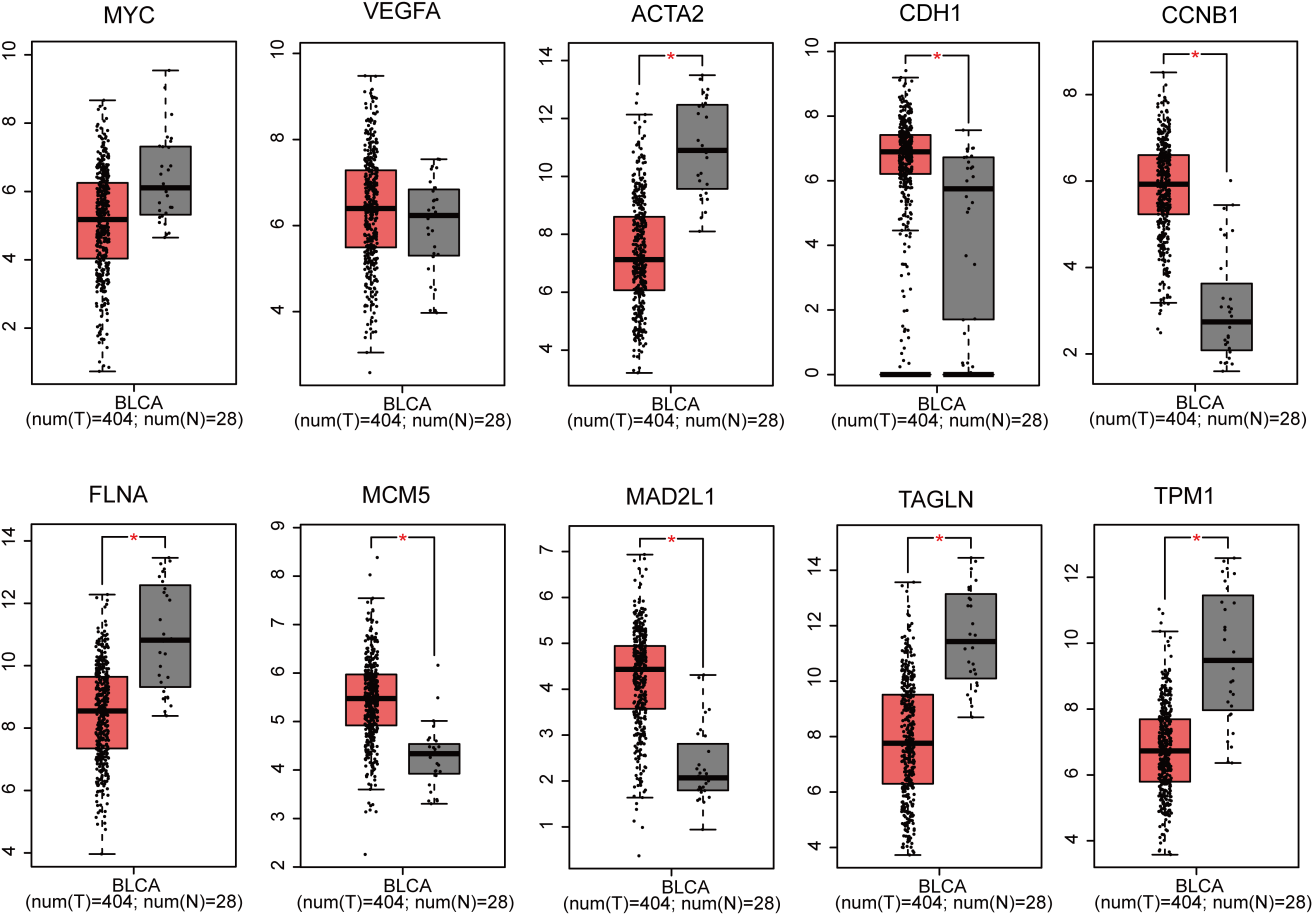
Gene expression of 10 genes (MYC, VEGFA, ACTA2, CDH1, CCNB1, FLNA, MCM5, MAD2L1, TAGLN and TPM1) based on GEPIA (*p < 0.05).

Utilizing GEPIA, the expression profiles of the ten genes (MYC, VEGFA, ACTA2, CDH1, CCNB1, FLNA, MCM5, MAD2L1, TAGLN and TPM1) in BLCA tissues and normal tissues was carried out. The findings revealed that the expression of MYC and VEGFA in BLCA samples was not substantially different from that of normal samples. Nevertheless, the expression of CDH1, CCNB1, MCM5 and MAD2L1 markedly up-regulated in BLCA tissues compared with normal tissues, and that of ACTA2, FLNA, TAGLN and TPM1 was significantly down-regulated (P<0.05 for all, **Figure 3**).

**Figure 4:**
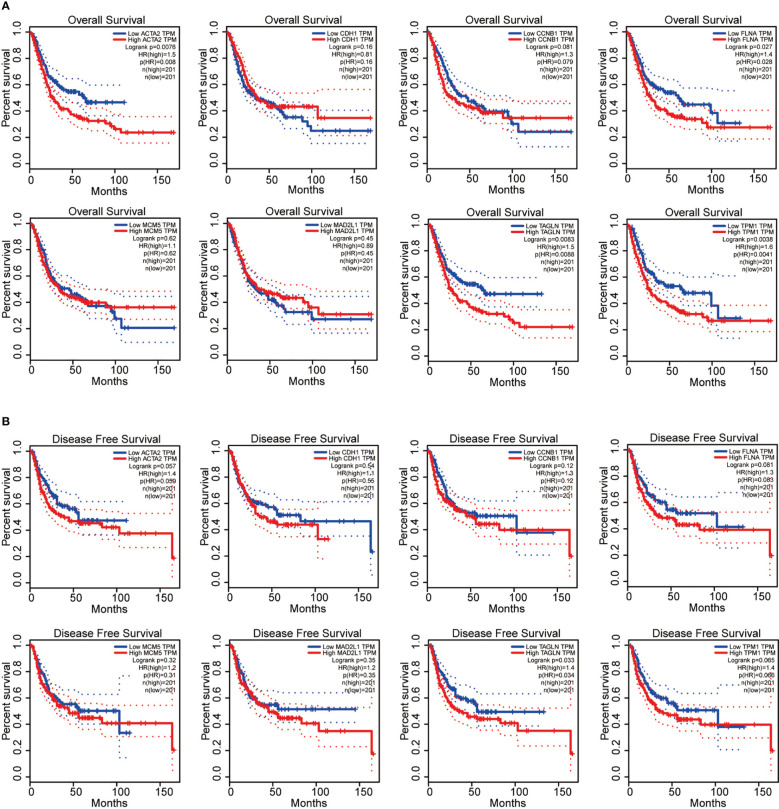
ACTA2, FLNA, TAGLN and TPM1 were correlated with the overall survival of bladder cancer **(A)**; TAGLN was associated with disease-free survival of bladder cancer **(B)**.

Next, we performed the survival analysis of these eight genes (ACTA2, CDH1, CCNB1, FLNA, MCM5, MAD2L1, TAGLN and TPM1) in bladder cancer. Among the 8 genes, ACTA2, FLNA, TAGLN and TPM1 were correlated with the overall survival of BLCA (**Figure 4A**), in comparision TAGLN was associated with disease-free survival of bladder cancer (**Figure 4B**). High expression of these four genes means a worse prognosis. In addition, CDH1, CCNB1, MCM5 and MAD2L1 were not significantly associated with the survival of patients. We verified the relationship between these genes and patient survival on the Kaplan Meier plotter website (**Supplementary Figure 1**).

### 3.4 Correlation analysis

**Figure 5:**
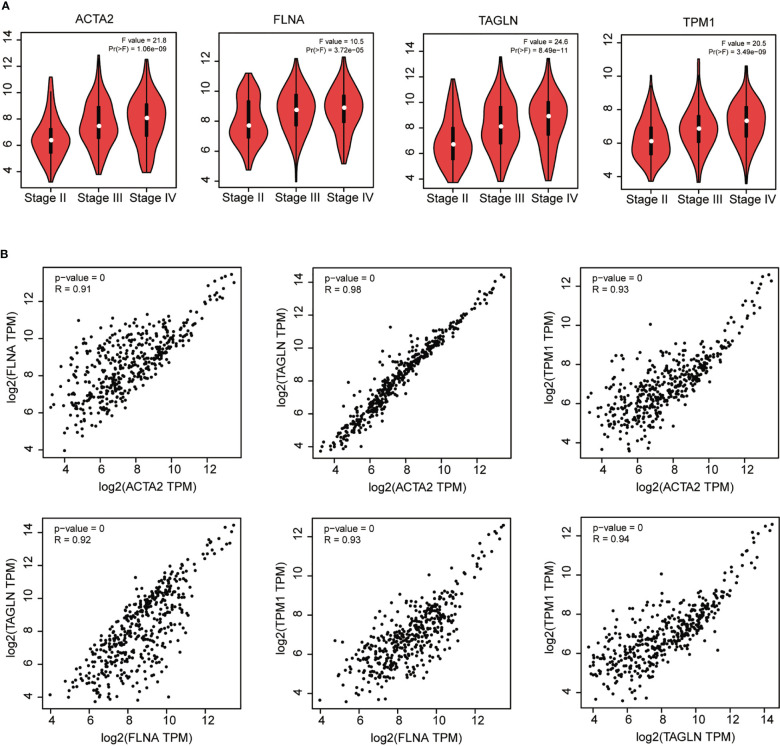
Correlation analysis of ACTA2, FLNA, TAGLN and TPM1 with the pathological stage of bladder cancer **(A)**. Correlation analysis between ACTA2, FLNA, TAGLN and TPM1 in bladder cancer **(B)**.

We found that ACTA2, FLNA, TAGLN, and TPM1 had a strong association with the pathological stage of BLCA by utilizing GEPIA (*p* < 0.05) (**Figure 5A**). Furthermore, there was a strong connection between the expression of these four genes: ACTA2 and FLNA (R = 0.91), ACTA2 and TAGLN (R = 0.98), ACTA2 and TPM1 (R = 0.93), FLNA and TAGLN (R = 0.92), FLNA and TPM1 (R = 0.93), TAGLN and TPM1 (R = 0.94) (**Figure 5B**).

### 3.5 Relationship between the candidate genes and related clinical parameters and their potential in diagnosis of bladder cancer

**Figure 6:**
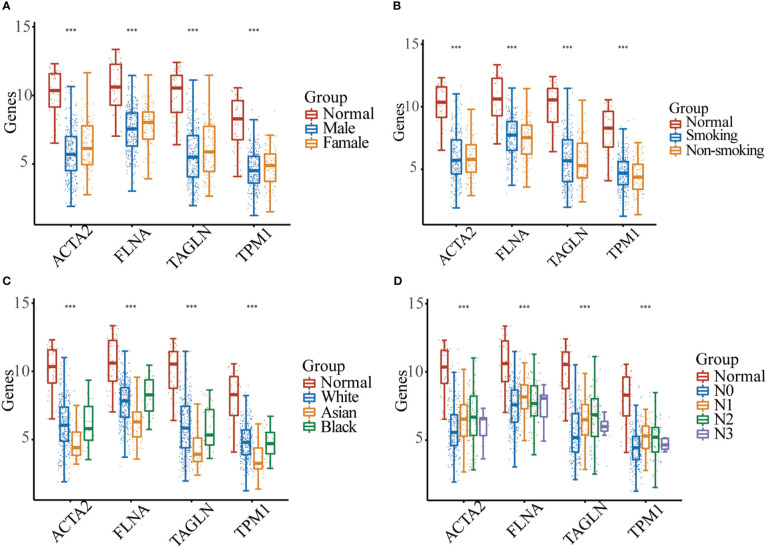
Expression analysis of four genes between normal samples and bladder cancer sample with different gender **(A)**, smoking habit **(B)**, race **(C)**, or the lymph node metastasis stage **(D)** (*** *p* < 0.001).

**Figure 7:**
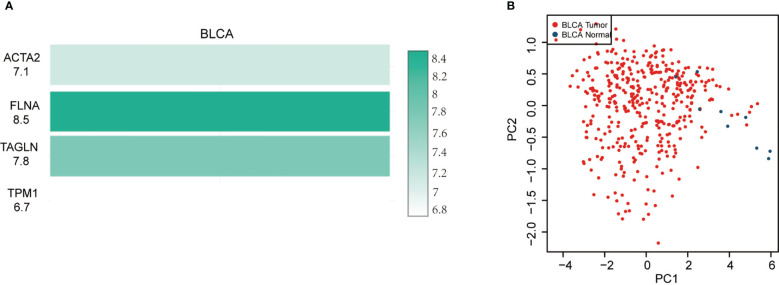
Four gene comparison analysis **(A)** in bladder cancer (BLCA), FLNA was the highest, followed by TAGLN, ACTA2 and TPM1. Principal components analysis (PCA) of ACTA2, FLNA, TAGLN, and TPM1 **(B)**.

In BLCA tissues relative to normal tissues, ACTA2, FLNA, TAGLN and TPM1 were significantly down-regulated in either gender, different races, smoker or non-smoker and various lymph node metastasis stages (**Figure 6**). Using GEPIA, the multiple-gene comparison of the four candidate genes for bladder cancer biomarkers was performed. FLNA was the most highly expressed, followed by TAGLN, ACTA2 and TPM1 (**Figure 7A**). The analysis of principal components (PCA) performed using TCGA data and GTEx data. We discovered that the four genes could successfully differentiate between BLCA tissues and normal tissues, suggesting the potential for the diagnosis of bladder cancer (**Figure 7B**).

### 3.6 Verification of expression of ACTA2, FLNA, TAGLN and TPM1

**Figure 8:**
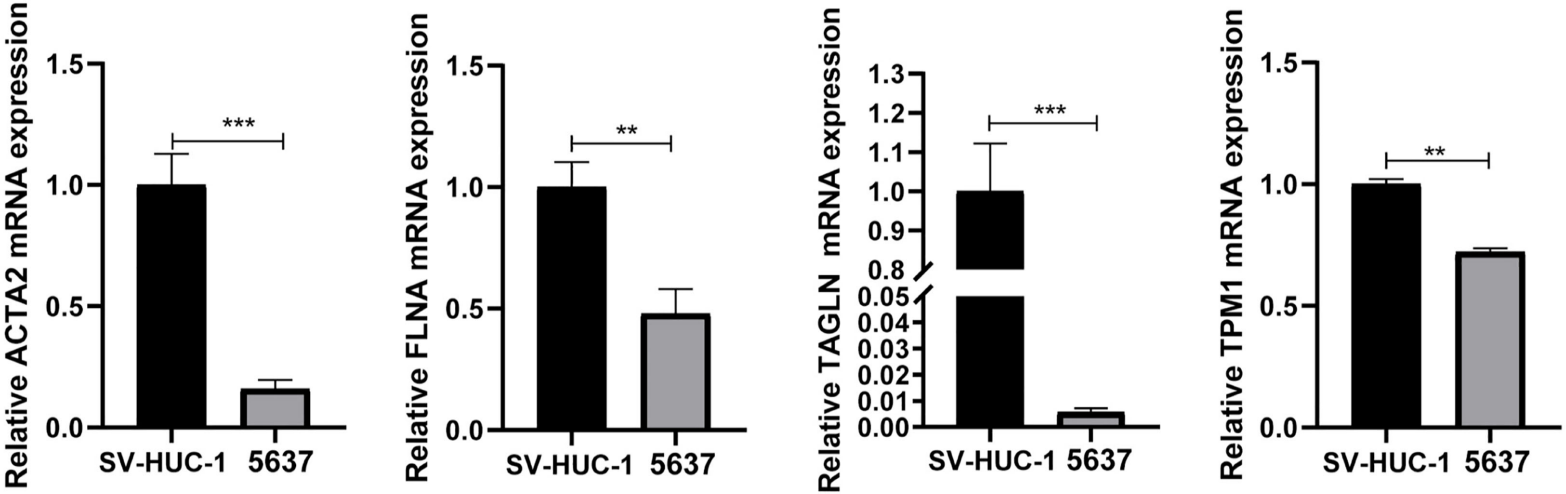
The mRNA was extracted from SV-HUC-1and 5637 cells, and the expression of ACTA2, FLNA, TAGLN, TPM1 was measured by qRT-PCR. The mRNA expression of ACTA2, FLNA, TAGLN, TPM1 was significantly decreased in 5637 cells (** *p* < 0.01; *** *p* < 0.001).

**Figure 9:**
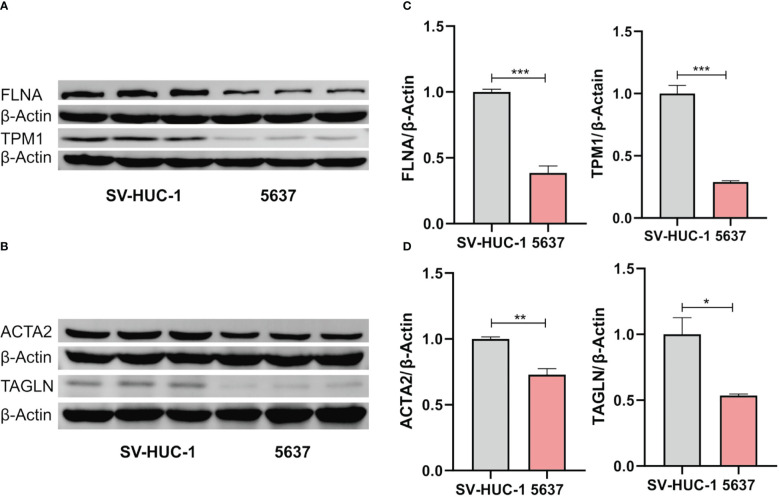
Western blot analysis of the expression of FLNA and TPM1 **(A)**, ACTA2 and TAGLN **(B)**. **(C, D)** Quantification analysis of FLNA, TPM1, ACTA2 and TAGLN proteins. The protein expression of ACTA2, FLNA, TAGLN and TPM1 was significantly decreased in 5637 cells (*p < 0.05; **p < 0.01; ***p < 0.001).

qRT-PCR demonstrated that the mRNA expression levels of ACTA2, FLNA, TAGLN, and TPM1 in bladder tumor cells were significantly decreased compared with normal bladder cells (**Figure 8**), which corresponded to the results of our prior bioinformatic investigation using public datasets. Similarly, Western blotting also confirmed that the protein expression significantly decreased in bladder cancer cells (**Figure 9**). Bladder cancer samples can be distinguished from normal bladder samples by detecting the expression of mRNA and protein of four genes, indicating the potential of four biomarkers in the diagnosis of bladder cancer.

### 3.7 Infiltration of immune cells

**Figure 10:**
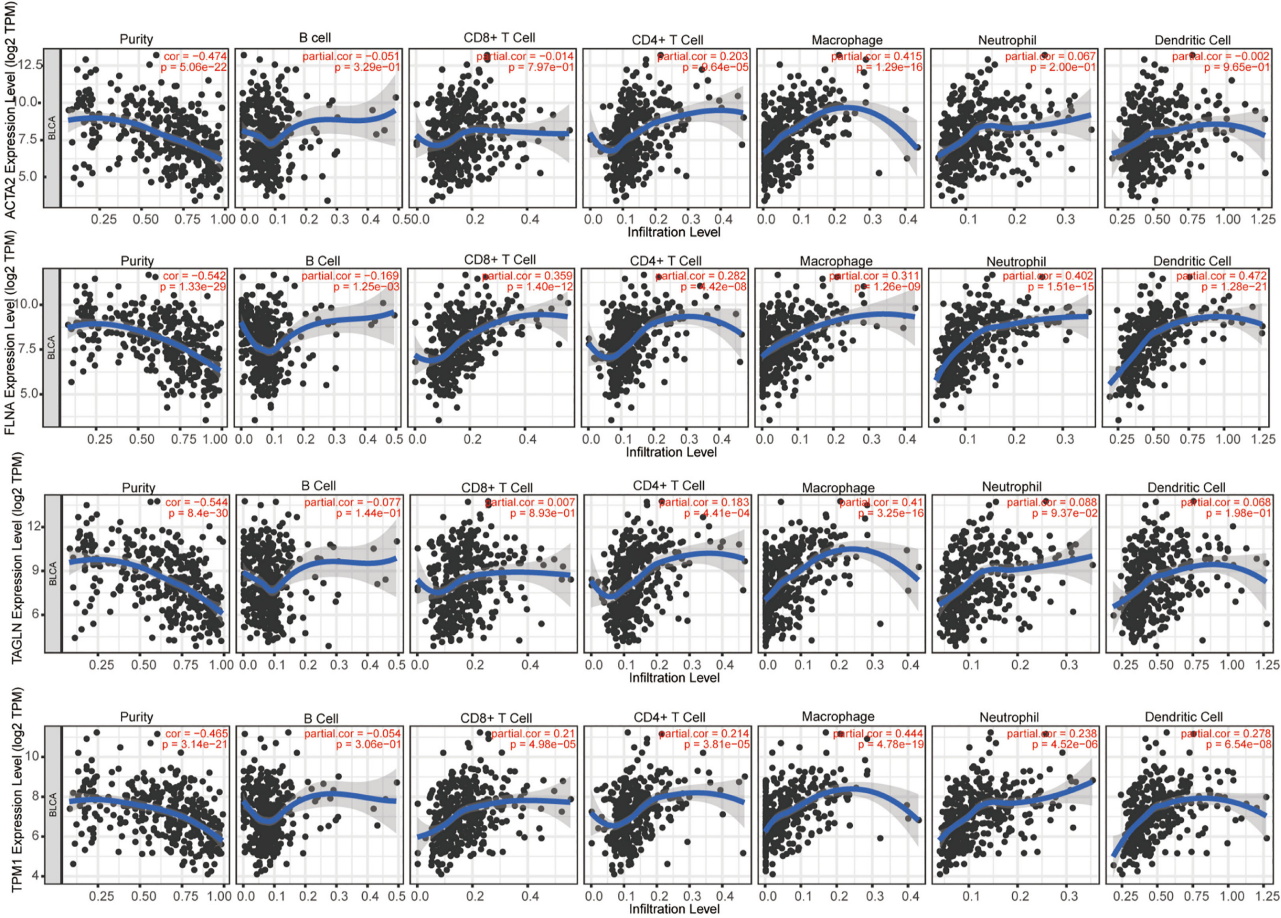
The correlation between ACTA2, FLNA, TAGLN, TPM1 with infiltration of different immune cells in bladder cancer analyzed by TIMER.

We evaluated the connection between four genes and several forms of immune cell infiltration in bladder cancer (**Figure 10**). r > 0.3 was defined as having correlation, and the expression of ACTA2 was positively linked with macrophage (r=0.415, p=1.29e-16), that of TAGLN (r=0.41, p=3.25e-16) and TPM1 (r=0.444, p=4.78e-19) positively with macrophages, FLNA was positively correlated with four types of cells: CD8+T cell (r=0.359, p=1.40e-12), macrophage (r=0.311, p=1.26e-09), neutrophils (r=0.402, p=1.51e-15), and dendritic cells (r=0.472, p=1.28e-21).

**Figure 11:**
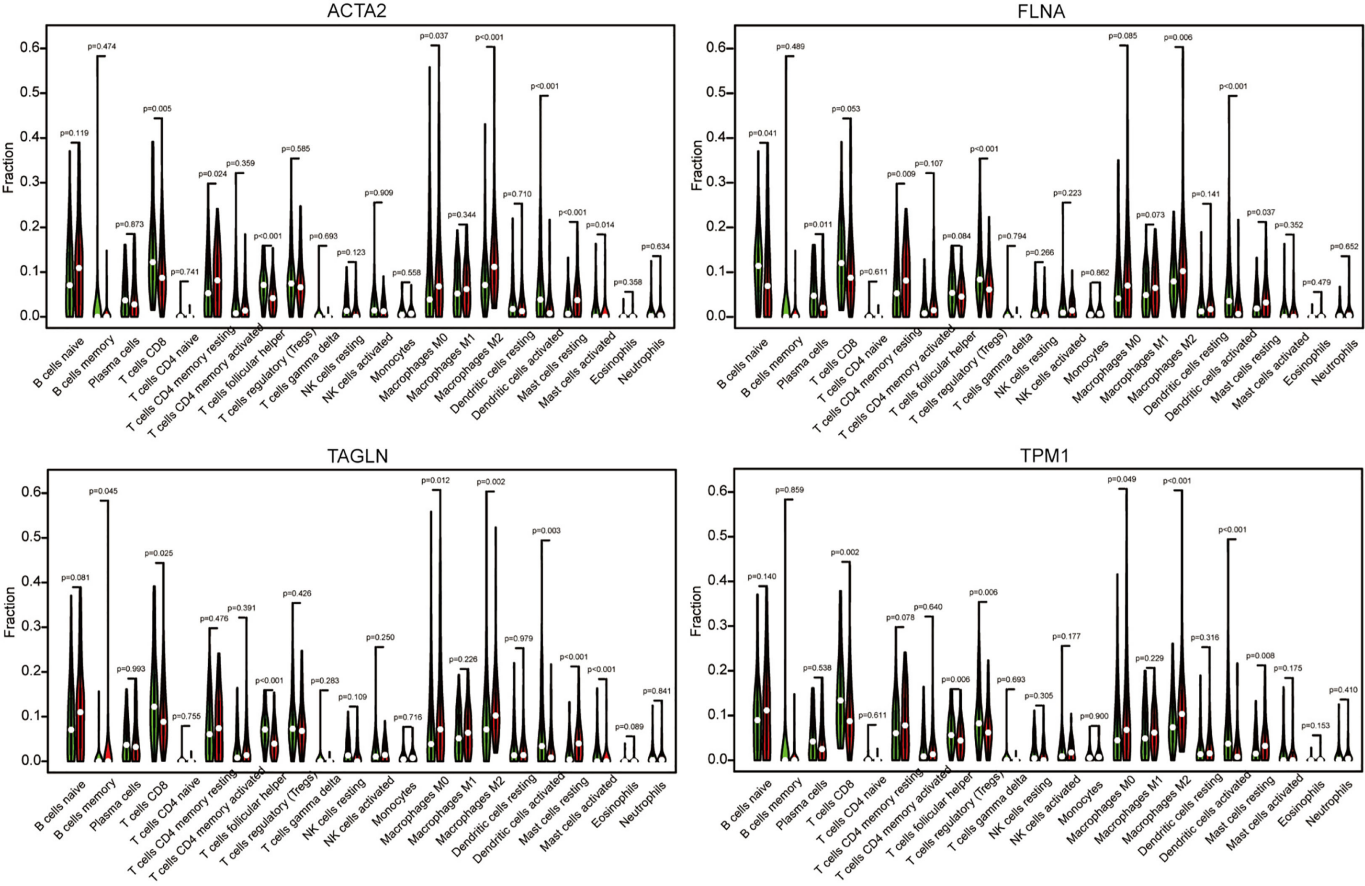
The cancer samples were divided into high expression group grand and low expression group based on the expression of ACTA2, FLNA, TAGLN, and TPM1. The group of low expression was shown in green, and the group of high expression was shown in red.

Using algorithm CIBERSORT, we obtained a more detailed immune cell infiltration profile. Further, we clarified the correlation between different immune cells and four genes (**Figure 11**). We discovered that the infiltration abundance of macrophage M2 was dramatically increased in the group with high ACTA2 (p<0.001), FLNA (p<0.001), TAGLN (p=0.002) and TPM1 (p<0.001) expression compared with the low expression group, which demonstrated a clear correlation between the amount of M2 infiltration and the expression of the four genes in the immune microenvironment of bladder cancer.

### 3.8 Analysis of co-expressed genes

**Figure 12:**
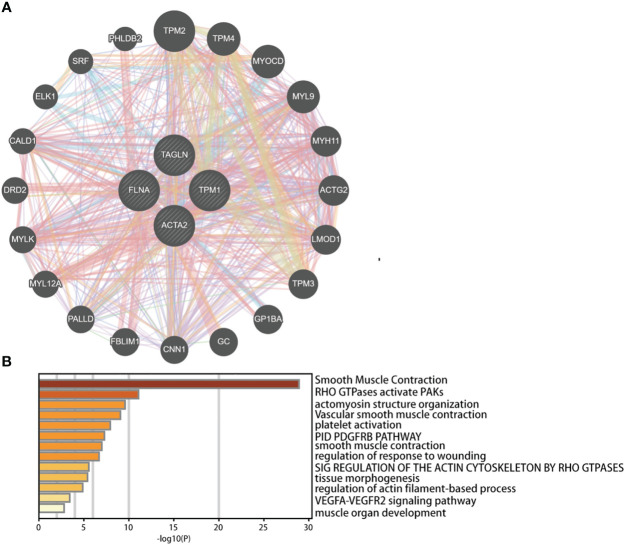
Predicted functions and pathways of ACTA2, FLNA, TAGLN, and TPM1 and co-expressed genes in bladder cancer analyzed by GeneMANIA **(A)** and Metascape **(B)**.

We investigated the co-expressed genes of these four genes (**Figure 12A**). We also conducted pathway analysis and functional enrichment of the co-expressed genes (**Figure 12B**). The co-expressed genes included TPM2, TPM4, MYOCD, MYL9, MYH11, ACTG2, LMOD1, TPM3, GP1BA, GC, CNN1, FBLIM1, PALLD, MYL12A, MYLK, DRD2, CALD1, ELK1, SRF, PHLDB2. The enrichment pathways were smooth muscle contraction, RHO GTPases activate PAKs, actomyosin structure organization, vascular smooth muscle contraction, platelet activation, PID PDGFRB pathway, smooth muscle contraction, regulation of response to wounding, sig regulation of the actin cytoskeleton by RHO GTPases, tissue morphogenesis, regulation of actin filament-based process, VEGFA-VEGFR2 signaling pathway, muscle organ development.

### 3.9 Validation of the relationship of TPM1 and FLNA to macrophages M2

**Figure 13:**
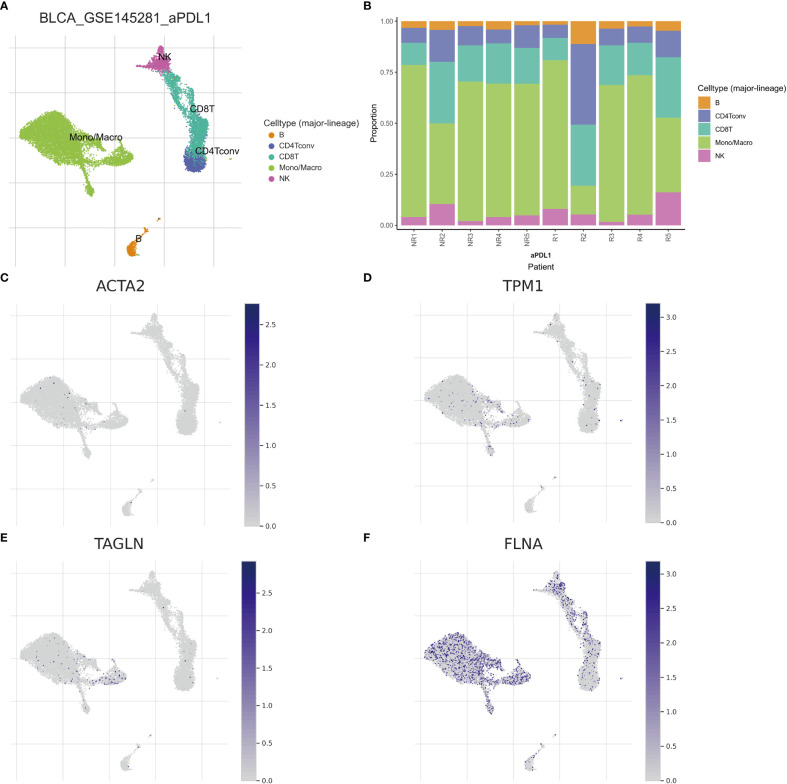
Different subtypes of immune cells in bladder cancer tissue. **(A)** Classification of immune cells by single-cell sequencing **(B)** Abundance of immune cells in each patient sample **(C–F)** Expression of ACTA2, FLNA, TAGLN, and TPM1 per immune cell.

**Figure 14:**
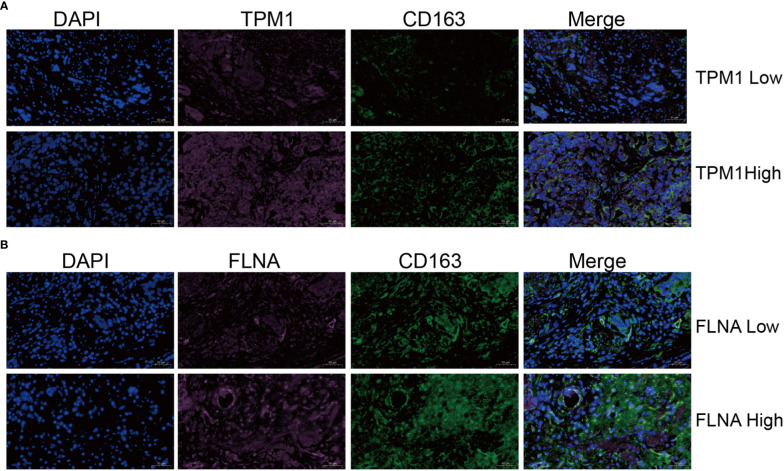
Validation of the relationship of TPM1 and FLNA to macrophages M2. **(A, B)** Multiple immunohistochemistry (mIHC) results showed that TPM1 and FLNA were positively correlated with CD163.

Single-cell sequencing examination of bladder cancer tissue revealed that cancer tissue includes a substantial number of B cells, NK cells, CT8 cells, CT4 cells, and monocyte-macrophages, with monocyte-macrophages being the most prevalent (**Figures 13A, B**). FLNA, TPM1, TAGLN, and ACTA2 were expressed at higher levels in monocyte-macrophages compared to other immune cells (**Figures 13C–F**). This conclusively demonstrated that the four genes were intimately associated with monocyte-macrophages. The ranking of the expression levels of four genes in monocyte-macrophages is FLNA, TPM1, TAGLN and ACTA2 from high to low. We selected FLNA, TPM1, and the M2 macrophage marker CD163 to perform mIHC on BLCA pathological specimens to verify their potential significance (**Figures 14A, B**). The findings revealed that the expression of FLNA, TPM1 was favorably connected with CD163, indicating that FLNA, TPM1 was positively correlated with M2 macrophages. These preliminary results validate our bioinformatics findings that FLNA and TPM1 play a role in M2 macrophage polarization.

## 4 Discussion

Bioinformatic analyses demonstrated that the expression of ACTA2, FLNA, TAGLN and TPM1 in bladder cancer was markedly downregulated relative to nearby normal tissue. They were correlated with the overall survival rate for bladder cancer, and TAGLN also correlated with bladder cancer disease-free survival. By survival analysis, we found that their overall survival and disease-free survival curves also showed similarities, such as ACTA2 and TAGLN, FLNA and TPM1, suggesting that these genes have similar functions and are likely to be co-expressed and play a synergistic role in bladder cancer. We further compared the correlations among the four genes and found that the correlations among them were all greater than 0.9, showing a strong correlation, especially the correlation coefficient between ACTA2 and TAGLN, which reached 0.98. Itfurther confirms that these genes have similar functions and play a synergistic role in bladder cancer. In the high expression group of ACTA2, FLNA, TAGLN, and TPM1, macrophage M2 infiltration abundance was dramatically increased compared to the low expression group. In addition, qRT-PCR and Western blotting demonstrated that the transcription and translation levels of ACTA2, FLNA, TAGLN, and TPM1 were much lower in bladder tumor cells than in normal bladder cells. The correlation analysis revealed a significant link between ACTA2, FLNA, TAGLN, and TPM1, implicating that ACTA2, FLNA, TAGLN and TPM1 might work synergistically in cellular processes. All these findings suggested that ACTA2, FLNA, TAGLN and TPM1 were potential diagnostic biomarkers for bladder cancer. The 4-gene biomarker could be used for the prognostic evaluation of bladder cancer.

FLNA, TAGLN and TPM1 are all members of the actin-binding protein family (29–31). They could combine ACTA and perform an important function in the formation of the cytoskeleton, vascular development, intercellular junctions, interstitial migration and other processes. Recent studies have shown that immune cells of tumor microenvironment were associated with tumor progression and recurrence and affected tumor response to immunotherapy. In this study, we observed that FLNA has a substantial positive correlation with CD8+T cell, macrophage, neutrophile, and dendritic cell; ACTA2, TAGLN and TPM1 had a positive correlation with macrophage. It suggested that these four genes, especially FLNA, reflect the immune status in bladder tumor microenvironment. We conducted a co-expression analysis of these four genes, and alsoconducted functional enrichment and pathway analysis of the obtained co-expressed genes. These co-expressed genes shown to be involved in several cellular functions and pathways. Some pathways were involved in tumor formation, such as the VEGFA-VEGFR2 pathway, which could promote angiogenesis and activate various signal transduction pathways such as ERK1/2, PI3K-Akt/PKB and phospholipase C-γ pathway (32), and plays an important part in the development of several malignancies, including bladder cancer.

Our investigation found that ACTA2, FLNA, TAGLN, and TPM1 substantially downregulated in BLCA samples compared to normal samples, independent of gender, race, or smoking behavior. This finding was consistent with recent publications (33, 34). The result of multiple-gene comparison and principal component analysis indicated that bladder cancer tissue and bladder normal tissue could be distinguished by the expression of the four genes, and this result had been verified by qRT-PCR and Western blotting. It is believed that ACTA2, FLNA, TAGLN, and TPM1 will serve as brand-new markers for the identification of BLCA. In the meanwhile, we investigated the ability to predict the prognosis of BLCA. The survival research revealed a correlation between ACTA2, FLNA, and TPM1 and overall survival. The prognosis of patients worsens with increased expression of these four genes. This finding suggested that the four genes may accelerate the formation of BLCA, which contradicted by the fact that the expression of the four genes was dramatically downregulated in bladder cancer cell. We discovered a clear correlation between macrophage M2 and four genes in bladder cancer. Previous studies showed that macrophages can be polarized from antitumor type (M1) to tumorigenic type (M2) (35), and M2 may stimulate the development and invasion of several cancers, including bladder cancer (36). Therefore, we speculated that the poor prognosis of the high-expression group might be related to the increase of M2 in the tumor microenvironment. Increased expression of ACTA2, FLNA, TAGLN, and TPM1 may promote the transformation of macrophages into M2 in the tumor microenvironment. This explains the poor prognosis of patients with elevated expression of ACTA2, FLNA, TAGLN, and TPM1. We hypothesize that locally targeted drugs that inhibit the expression of these four genes may reduce macrophage M2 polarization and enhance systemic responses via interactions with microenvironmental molecules, possibly causing abscopal effects and affecting tumors outside the treatment area.

In several malignancies, including pancreatic ductal adenocarcinoma and invasive breast cancer, ACTA2 has been related to tumor-associated fibroblasts, and has a function in cell shape, cellular integrity, and motivation. It may influence tumor invasion and metastasis, which was confirmed in Lee’s study (37). Down-regulation of ACTA2 dramatically decreased lung adenocarcinoma cell movement and invasion. The mechanism may be that ACTA2 stimulates the release of IL-6 from tumor-associated fibroblasts (CAF), induces epithelial-mesenchymal transformation (EMT), and induces the transformation from non-invasive tumor type to invasive tumor type (38). In our study, patients with higher ACTA2 expression had worse prognoses and lower survival rates.

FLNA is a cross-linking protein of actin. Its functions include cell signaling, protein degradation, transcriptional regulation, etc. It allows FLNA to participate in tumor growth and invasion (39). This involvement enables FLNA to play a role in tumor proliferation and invasion. FLNA exhibits two distinct effects depending on its cell localization. High levels of FLNA in the cytoplasm are associated with invasive tumors. In the nucleus, FLNA is involved in transcription factor interactions to limit tumor invasiveness (40). Our study showed that FLNA expression was reduced in bladder cancer, but it was not clear whether this happened in the cytoplasm or the nucleus.

TAGLN is an actin-binding protein that may modulate the actin cytoskeleton, regulating cancer cell proliferation and migration. It is mainly expressed in the cytoplasm, not in the nucleus, where it binds to F-actin (41). Earlier investigations have shown that TAGLN is an antitumor gene with decreased expression in BLCA (42), and its overexpression inhibits bladder cancer cell growth, invasion, and migration. However, additional research has shown that TAGLN has carcinogenic properties and is overexpressed in bladder cancer (43), promoting tumor progression and metastasis. This disparity showed that the function of TAGLN in the development, proliferation, and metastasis of BLCA remains ambiguous and requires additional investigation. Our results showed that TAGLN was down-regulated in BLCA cells, supporting that TAGLN is an anti-tumor gene.

TPM1 encodes an actin-binding protein which is important for the contraction of striated and smooth muscle as well as the formation of the cytoskeleton. It is a tumor suppressor gene. According to the research completed so far, it plays a function in lymphatic metastasis and prevents the progression of bladder cancer (44).

There are some limitations in our study. To evaluate and investigate the particular processes through which ACTA2, FLNA, TAGLN, and TPM1 contribute to the progression of BLCA, more *in vitro* and *in vivo* investigations and clinical samples are required. In addition, whether the expression levels of these four genes are consistent in NMIBC and MIBC requires further investigation.

## 5 Conclusions

Our study demonstrated that ACTA2, FLNA, TAGLN, and TPM1 were associated with patient survival. qRT-PCR and Western blotting demonstrated the ability to distinguish bladder cancer samples from normal samples. Four genes affect the immune cells’ infiltration of the tumor microenvironment, providing a new direction for tumor immunotherapy. In the future, these four genes may serve as crucial indicators for the clinical diagnosis and prognostic assessment of bladder cancer.

## Data availability statement

Publicly available datasets were analyzed in this study. This data can be found here: GEO database (https://www.ncbi.nlm.nih.gov/geo/) and TCGA database (https://portal.gdc.cancer.gov/), the accession numbers are GSE3167 and GSE188715.

## Author contributions

Study conception and design worked by HL, material preparation, data collection and analysis were performed by XLL, XXL and QK. The first draft of the manuscript was written by XLL and XXL. HL revised the manuscript. All authors read and approved the final manuscript.
