# Supplementary material for: Screening of immunotherapy-related genes in bladder cancer based on GEO datasets
Source: Front Oncol. 2023 May 18;13:1176637. doi: 10.3389/fonc.2023.1176637 (PMC10232963; doi:10.3389/fonc.2023.1176637)
Supplement: Supplementary file 1 [file Table_1.docx]

Supplementary Material

## Supplementary Figures


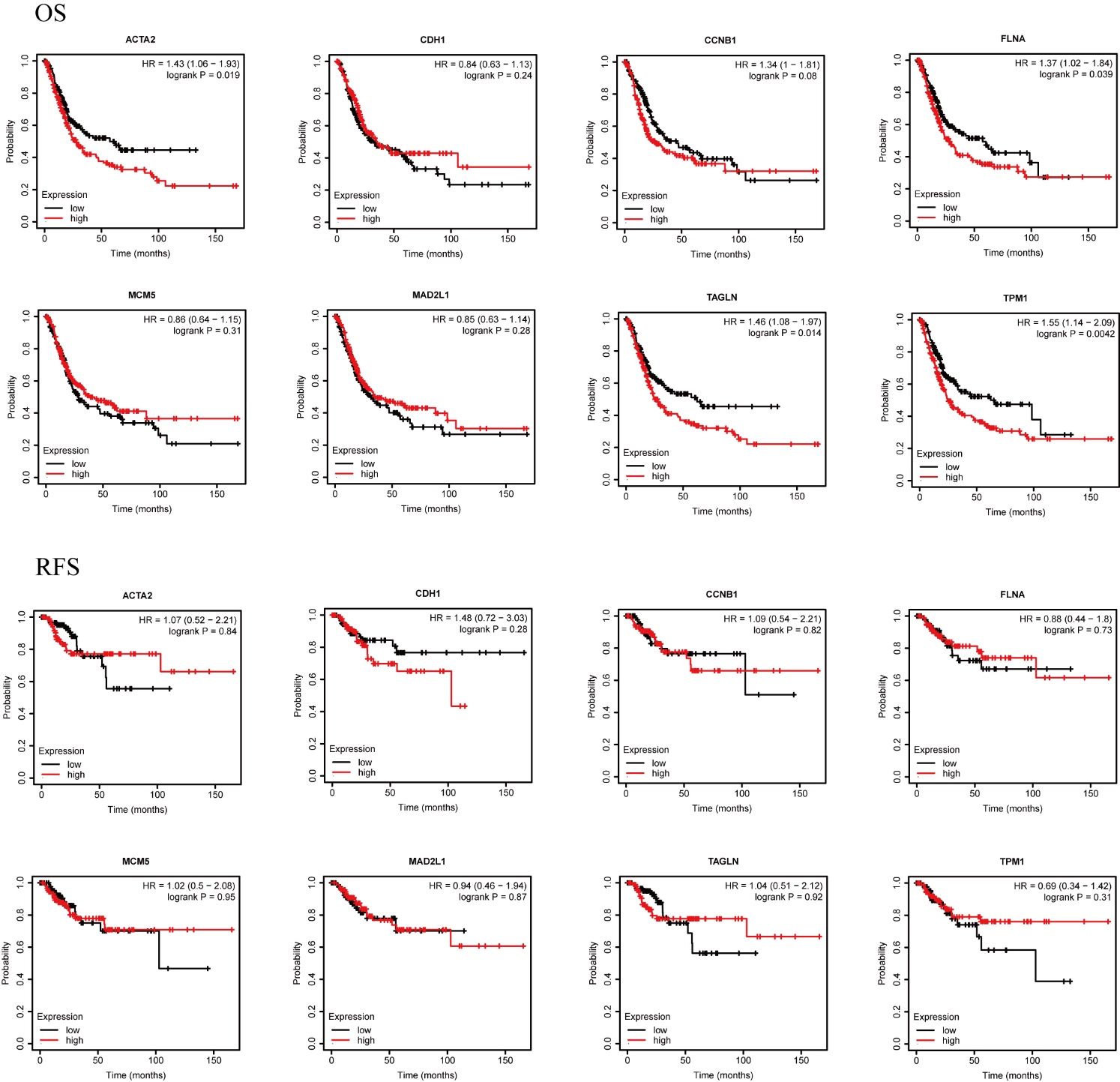


**Supplementary Figure 1.** The result of overall survival (A) and disease-free survival (B) analysis of 8 candidate genes (ACTA2, CDH1, CCNB1, FLNA, MCM5, MAD2L1, TAGLN and TPM1) by the Kaplan-Meier Plotter.
